# Learning from returnee Ethiopian migrant domestic workers: a qualitative assessment to reduce the risk of human trafficking

**DOI:** 10.1186/s12992-017-0293-x

**Published:** 2017-09-11

**Authors:** Joanna Busza, Sehin Teferra, Serawit Omer, Cathy Zimmerman

**Affiliations:** 1 Department of Population Health, London School of Hygiene & Tropical Medicine, Keppel Street, London, WC1E 7HT UK; 2 Independent Research Consultant, Addis Ababa, Ethiopia; 3 Department of Global Health and Development, London School of Hygiene & Tropical Medicine, Keppel Street, London, WC1E 7HT UK

**Keywords:** Migration, Ethiopia, Domestic work, Qualitative research, Risk reduction

## Abstract

**Background:**

International migration has become a global political priority, with growing concern about the scale of human trafficking, hazardous work conditions, and resulting psychological and physical morbidity among migrants. Ethiopia remains a significant “source” country for female domestic workers to the Middle East and Gulf States, despite widespread reports of exploitation and abuse. Prior to introduction of a “safe migration” intervention, we conducted formative research to elicit lessons learned by women who had worked as domestic workers abroad. The aim of the study was to identify realistic measures future migrants could take to protect themselves, based on the collective insights and experience of returnees.

**Methods:**

We conducted a qualitative assessment among returnee domestic labour migrants in Amhara Region, Ethiopia, an area considered a “hotspot” for outmigration. We conducted in-depth interviews and focus group discussions with a total of 35 female returnees, exploring risk and protective factors experienced by Ethiopian women during domestic work abroad. We used thematic content analysis to identify practical messages that could improve prospective migrants’ preparedness.

**Results:**

Returnees described the knowledge and skills they acquired prior to departure and during migration, and shared advice they would give to prospective migrants in their community. Facilitators of positive migration included conforming to cultural and behavioural expectations, learning basic Arabic, using household appliances, and ensuring safety in employers’ homes. Respondents also associated confidence and assertiveness with better treatment and respect, and emphasized the importance of access to external communication (e.g. a mobile phone, local sim card, and contact details) for help in an emergency. Following their own challenging or even traumatic experiences, returnees were keen to support resilience among the next wave of migrants.

**Conclusions:**

There is little evidence on practices that foster safer migration, yet attention to human trafficking has led to an increase in pre-migration interventions. These require robust evidence about local risk and protective factors. Our findings identify knowledge, skills, attributes and resources found useful by returnee domestic workers in Amhara region, and have been used to inform a community-based programme aiming to foster better decision-making and preparation, with potential to offer insights for safer migration elsewhere.

## Background

Migration has become one of the most significant political, social and economic issues of our time, and concern about large scale population flows from the global south to wealthier countries dominate media attention and policy debate. Although accurate figures on the prevalence of international migration are impossible to determine, the International Labour Organization (ILO) posits that among the estimated 244 million cross-border migrants, 150 million are labour migrants, defined as motivated by economic opportunities [1].

Female labour migrants commonly take up domestic work, often operating in unregulated or illegal markets [2]. The ILO reports that there are 11.5 million migrant domestic workers and over three quarters are female [1]. The health of labour migrants intersects with their work conditions and exposure to risks both during transit and at destination [3]. Domestic labour and other “caring” roles have been associated with various hazards including risk of injury [4], exposure to toxic chemicals [5], sleep deprivation [6], stress and other mental health problems [7], and physical and sexual abuse [8]. Furthermore, migrant domestic workers risk being trafficked if they are unable to access proper channels for legal entry and employment, which further carries significant health and safety risks [3, 9, 10].

Ethiopia is a large “source” country for female domestic workers to the Middle East and Gulf States [11]. An estimated 170-180,000 women depart each year among whom 60-70% are estimated to be irregular migrants [12]. Conditions for Ethiopian workers abroad are marked by frequent exploitation due to weak labour laws and regulations and the fact that many women procure work on tourist visas or through other irregular means, reducing any access they may have to redress for abuses [13]. Exploitation, neglect, physical and sexual abuse against Ethiopian domestic workers have been documented [14–16]. A recent survey of 1036 migrants returning from the Middle East and South Africa found a high burden of mental health problems resulting from adverse migration experiences, with 27.6% respondents reporting symptoms of common mental disorders [17].

In response, the Ethiopian government has adopted a policy of trying to discourage labour migration and conducts awareness raising campaigns intended to dissuade potential migrants by emphasising the dangers of human trafficking [18]. Concerns about the welfare of Ethiopians abroad culminated in 2013 when Saudi Arabia forcibly deported 160,000 undocumented Ethiopian migrants and the Ethiopian government responded by legislating an outright ban on labour out-migration [19]. Although the ban was lifted in late 2015, preventing out-migration remains the focus of many programmes [20].

To provide alternative messages about safe migration, the donor organisation The Freedom Fund is supporting a new initiative called the “Hotspot” programme. It aims to reduce the risk of human trafficking in domestic work abroad by targeting prospective migrants, returnees considering re-migration, their families and communities. The 3-year programme launched in 2015 and will work through mid-2018 in two sites, the area between Dessie and Hayk towns in Amhara region and Addis Ababa Ketama sub-city. These locations were selected for their high numbers of departing migrants. Amhara is a large rural region from which many female migrants originate, while Ketama sub-city is the area surrounding the capital city’s bus station, where many migrants arrive first to make further arrangements or take up interim domestic work in Ethiopian households.

The focus of the programme is to encourage better preparedness for safe migration [21]. The programme is funding 14 local implementing partners to raise awareness in schools, community associations, and through local media of specific risks that are confronted pre-departure, during transit, and at destination, and to provide practical information on how to prevent or mitigate these. Vocational training for returnee domestic workers in business development is also being offered to reduce the likelihood that women will be trafficked to pay back existing debts from their earlier migration.

In order to identify appropriate messages about migration risks and develop evidence-based recommendations for how to improve migration safety, the Freedom Fund commissioned formative research among returnees from the Gulf States in their Amhara programme site. The aim of this study was to elicit lessons learned by women who had worked as domestic workers abroad, specifically their insights into what measures future migrants might realistically take to protect themselves. The findings were used to inform the development of the Amhara region Hotspot programme.

## Methods

We conducted a rapid qualitative assessment to inform design of intervention activities, particularly in terms of tailoring messages to prospective migrants about how to maximise their safety. The study took place in 4 *kebeles* (smallest Ethiopian administrative unit, roughly equivalent to a *ward*) of one *woreda* (district) located on the outskirts of Hayk town in Amhara region of Ethiopia, considered to be typical of the surrounding area in terms of socio-demographic profile, accessibility by road, and reliance on agricultures by most of the population.

Due to political sensitivities around migration following the 2013 government ban and some anxiety around the concept of “safe migration” being promoted over “ending migration”, we relied on cooperation with local authorities to recruit returnees. First, we held discussions with *woreda* authorities such as representatives from the local Labour and Social Affairs and Women and Children’s Affairs offices. With their assistance, we contacted returnee migrants.

Data collection took place in January and February 2016 by the second and third authors (independent research consultant and fieldwork assistant, respectively), both of whom are female Ethiopians and fluent in the local language (Amharic). Focus group discussions (FGD) brought together female returnees from diverse backgrounds and perspectives, e.g. a range of age groups, religions, education levels and migration experiences. Participants were grouped by age group, loosely divided into women either younger or older than 30. We recruited any women who had worked in domestic labour in the Gulf States and were willing to talk about their experiences. The fieldwork assistant spent 1-2 days in the field site prior to arranging FGD in order to make the relevant contacts, and ask identified returnees to invite other peers (snowball sampling). We held FGD in public venues such under the shade of a tree in residential neighbourhoods, and obtained verbal assent at the start. No compensation was provided to participants.

Interview respondents were selected from among FGD participants. Following FGD, women who had shared interesting information or appeared to have particularly strong opinions about safe and risky migration were invited for interview. Interviews were arranged for times and locations convenient to participants (usually their homes), and written informed consent was obtained. All interviews and group discussions were digitally recorded and translated into English by the fieldworker. Ethical approval for the Ethiopia Hotspot study was obtained from the London School of Hygiene and Tropical Medicine and Addis Ababa University.

We conducted in-depth interviews with 12 returnee migrant women and focus group discussions with 23 returnee migrants. This paper presents findings on facilitators and barriers to safer migration experiences, focusing on influences during transit and at destination. Table 1 summarises socio-demographic and migration-related variables for the 12 in-depth interview respondents. We did not collect personal information (except age) during FGDs, as these were designed to elicit group social norms, background information and help identify respondents for follow-up interviews.

**Table 1:** Characteristics of in-depth interview respondents

	Current age	Years of education	Religion	Marital status	Destination(s) & duration	Migration process	Current occupation/ Plans
1	24	7	Christian	Divorced	Qatar (18 months)	• Parents sold cattle to raise money for journey • Used Addis Ababa based broker	Unspecified
2	23	6	Muslim	Married	Dubai (30 months)	• Dropped out of school to work in Addis Ababa first • Borrowed money from relative • Used Addis Ababa based broker who sent her friend	Working in her own shop but planning to re-migrate as business not going well.
3	28	Illiterate	Muslim	Single	Saudi Arabia (22 months)	• Worked as maid in Addis Ababa • Heard about overseas work opportunities on TV	Working in her own shop and hopes business will succeed.
4	24	5	Muslim	Single	Dubai (29 months)	• Used local broker	Planning to re-migrate to Saudi Arabia as her mother spent all her earnings
5	25	9	Muslim	Married	Dubai (26 months) Saudi Arabia (Time not provided)	• Dropped out of school • Found broker in Addis Ababa through uncle • Used agency in Addis Ababa for second migration	Unspecified
6	29	8	Christian	Divorced	Saudi Arabia (36 months)	• Worked as maid in Addis Ababa • Used agency in Addis Ababa • Had to wait 7 months for visa	Unspecified
7	22	10	Muslim	Single	Dubai (Time not provided) Saudi Arabia (24 months)	• Used illegal Addis Ababa broker for first migration • Used legal agency for second migration	Plans to re-migrate as soon as possible
8	24	8	Muslim	Married	Saudi Arabia (24 months)	• Worked at relatives’ home in Addis Ababa • Used agency in Addis Ababa	Unspecified
9	22	10	Muslim	Married	Saudi Arabia (27 months)	• Used local broker	Owns a shop with her husband
10	20	6	Muslim	Divorced	Dubai (24 months) Qatar (3 months)	• Borrowed money from relatives for first migration • Used legal agency for first migration with contract • Used illegal local broker for second migration as ban was in place by then; he “sold” her to employers and took all the earnings	Unemployed and “bored”; has sent passport to a broker to try re-migration to Dubai
11	35	4	Christian	Divorced	Dubai (Time not provided) Saudi Arabia (Time not provided)	• Used local broker for first migration • Second time to Saudi Arabia, from description appears to be illegal although she did not specify	Engaged in farming
12	20	8	Muslim	Single	Dubai (24 months)	• Father made all the arrangements	Plans to re-migrate

The first and second authors read all transcripts and coded text thematically. As the study aimed to identify differences between “safe” and “risky” migration, analysis began with topics selected a priori i.e. women’s perceptions of what led to positive or negative migration experiences. We applied an existing conceptual framework that divides migration into five phases (pre-departure, transit, destination, interception and return), but concentrated on the first three to explore how migrants planned their migration and dealt with risks confronted while abroad [3]. We then coded text into sub-themes that emerged directly from the data, such as the role of God and luck, and importance of personal hygiene.

## Results

We identified three main themes relating to what migrants learned from their experiences that could usefully translate into messages to better prepare others for safe migration. These were (1) how migrants gathered and interpreted information prior to their departure, and whether they felt adequately prepared, (2) what risks migrants confronted while abroad and how they managed these, and (3) what advice returnees themselves believe to be most valuable for the prospective migrants in their communities.

Direct quotes from transcripts are provided to illustrate these themes. Excerpts from in-depth interviews with returnee migrants are identified by number corresponding to Table 1 while all FGD quotes provide age of respondent.

### Preparing to leave

Respondents described relying on previous returnee migrants for useful information about migration, as well as on the families of current migrants living overseas. Through informal conversations, women assembled a picture of the kind of work expected, potential difficulties with employers, and learned several Arabic words and expressions.

Respondents explained such conversations reduced their apprehension about the destination country and work conditions. They suggested that the tales of returnees normalised their upcoming experience. For example, two participants noted how they sought information about culture and salaries:

*Before I went to the destination country I tried to find out from the returnees about the culture of Arab society, how I can easily assimilate myself with their culture, how I can easily understand their behaviour and so on. [Interview 6]*

*We got information from the relatives of migrant women. … We asked them how much money migrant women sent and how much they received from the bank. Then we compared the money received from Saudi Arabia, Kuwait and Dubai, after that we discussed where we should go to get better money, we always discuss about these issues at school. [Interview 12]*

One woman described how she adapted to life in Dubai quickly thanks to information from a neighbour, while those who had sisters who migrated before them felt they had the advantage of personal tuition in relevant skills and basic Arabic. At the same time, however, some participants said they knew nothing before departure and were thus unprepared for their new situation, as indicated by one woman who explained:

*I just didn’t know what will happen to me. I said ‘okay’ to everything asked by my employers because I didn’t know my rights and duties, but now I have matured enough to know my duties and responsibilities, I would surely ask for my rights now. [Interview 2]*

More formal channels of information were mentioned, particularly government sponsored efforts to warn women of the dangers of trafficking, and pre-departure trainings offered through registered employment agencies. Returnees who had participated in trainings recalled variable quality, suggesting some were comprehensive and others perfunctory. Some recruitment agencies showed videos with basic information on work conditions, domestic skills, and adaptation to local customs (religion, dress, use of sanitary products), while others referred women to Ministry of Labour and Social Affairs workshops. Overall, returnees who received pre-departure training spoke highly of it and indicated they put their new knowledge into practice, for example by avoiding injury from strong cleaning solvents, separating dark and pale laundry, and correctly operating household appliances. One described the helpful content of her training, noting that it boosted her confidence:

*They can show you how you perform household chores … how to clean the house, and how to communicate with your employers. This would definitely help you to be aware of your responsibilities in the destination country …and it makes you confident. [Interview 7]*

Public campaigns warning against working abroad were generally viewed with derision. While community members did not question the validity of information reported through public education initiatives, warnings did not seem to outweigh the positive narratives in circulation. For some, their intention to migrate seemed irreversible:

*If you ask me, I won’t change my mind if I heard some woman died in Arab countries ... Let me tell you I went to Dubai immediately after one woman died from the neighbourhood. My mother tried to stop me from going, but I said ‘no’. Nothing happened to me. [Interview 10]*

Another woman concurred with the ineffectiveness of these campaigns:

*There are people from the woreda who provide advice especially for those who choose to travel by sea. However, people don’t want to listen to any of this advice. They totally reject it. … They just think of the money they will earn. [Interview 11]*

A third woman explained that prospective migrants seek out positive stories and discount reports of negative experiences. When planning her departure, she focused on the successes she saw among returnees and ignored “bad news”:

*I was motivated to go when I saw how women in the village who went to Arab countries changed their lives. I asked myself ‘Why don’t I go there and change my life just like them?’ … I saw them sending money to support their families. They also built houses and fulfilled their needs. … We saw them wearing gold. … We don’t like to hear their negative experiences. [Interview 1]*

The phrase “to change my life” was an oft-repeated refrain in women’s accounts of their motivation to migrate. Respondents described how they were totally fixated on the opportunities ahead and thus did not put much thought into how to prepare for adverse circumstances they might confront.

### Managing life abroad

Returnee women stressed that their real learning started on arrival, when they confronted the realities of domestic labour and the specific conditions of their employer’s household. As domestic workers, they were answerable to the woman of the house, who would train her new employee or ask another maid to do so:

*In my case my employer brought another Ethiopian maid from her mother’s house so that she can train me for three days. She showed me how to clean houses, how to cook their food, and how to wash and iron clothes. I kept away myself from the washing machine while she was showing me how to use it, I was so scared of the noise it made! [Interview 2]*

In addition to understanding household appliances and required tasks, it was important for domestic workers to adopt appropriate personal demeanour, dress and behaviour to ensure they did not displease employers.

*In my case I never saw my [male] employer ... When he arrived home he usually made a sound, and then I quickly went to the kitchen until he entered to his room. If they were in the living room and I wanted to prepare the dining table, I knocked on the door and they left the room until I finished, then they came back after I finished. … I wore a normal long skirt while I was working in the house. It’s only obligatory to cover your hair. But if we went out of the house we had to cover our whole body except our eyes. [FGD, 30]*

Many respondents mentioned that personal hygiene was important to local employers, particularly familiarity with disposable menstrual supplies, which was not universal among Ethiopian migrants.

*The Arabs consider Ethiopians as dirty. It is because some woman doesn’t know how to use sanitary pads, especially those who came from rural areas … They don’t know they should put it into garbage after they use it. Some of them put it in the latrine and it blocks the sewage system. [Interview 7]*

In terms of exposure to exploitation, in every interview and FGD, women reported having heard about or directly experienced abuse during their employment, including physical, sexual and psychological. This was usually attributed to “bad luck” in terms of having cruel employers, but returnee migrants also said they learned that specific behaviours or characteristics could reduce the likelihood of maltreatment, including being polite yet assertive, and expressing confidence:

*The Arabs respect a woman who has confidence and who knows her rights, they abuse you if they know that you don’t have confidence and if all the time you say ‘okay’ to them. [FGD, 31]*

*It is advisable to prepare yourself to be confident, not to get confused with what you see or hear. You have to convince yourself that you went there for work; you have to change your behaviour if it is necessary. If you are shy here you should no longer be shy there. Confident and strong characteristics helps you to be tolerant and successful. [Interview 6]*

One woman gave a very specific example of how calmly insisting on humane treatment changed her employer’s attitude to her:

*Sometimes they order you to eat their leftovers. At that time, I told her ‘I don’t want to eat that’, and then she gave me fresh food. Even I started preparing my meals for myself. You have to be confident and ask for what you want. [FGD, 20]*

Finally, the ability to communicate with people outside the employers’ household proved critical to migrants’ perceptions of safety and well-being. Ownership or access to a mobile phone was repeatedly mentioned, often as a source of conflict with employers, who were worried that the women might use the phones to arrange premature departure (i.e. before the end of the contract period, for which the employer would have paid brokerage fees, travel and visa costs, etc.). The ability to phone local friends or family members in Ethiopia signified personal freedom, independence and access to help if required. Trying to negotiate phone access was thus a key lesson from the migration experience.

*I usually called [home] three or four times a month because I have mobile phone. If I want someone’s phone number I asked my parents, and they searched and gave it to me when I called them back. [Interview 6, 29]*

*If you have mobile phone you can call the police or to the [employment] agency whenever you come across any challenges. If you don’t have a mobile phone how can you call someone to help you? Your employer can abuse you the way she wants. [Interview 7]*

Among respondents, however, being able to keep a phone was much rarer than being forbidden to have one, although employers generally allowed occasional use of their own phones, with a few exceptions.

*My employers didn’t allow me to have a mobile phone, but there are some who buy mobile phone for their maids. [Interview 1]*

### Advice for prospective migrants

We explicitly asked respondents to share information they felt could be of direct benefit to women considering migration abroad. Despite the ban, most returnees gave examples of how they already shared their experiences with others. Many highlighted, however, that as they themselves had focused on positive stories they had heard prior to departure, they found their own admonitions often ignored.

*When we tell our bad experiences to prospective migrants they don’t trust us. They usually say ‘Why do you forbid us while you went there and returned back with money?’ … Most of us are not convinced unless we experience it ourselves. [Interview 1]*

In response, returnees conveyed practical measures prospective migrants could take to improve their chances of having safe and successful migration experiences. These included learning Arabic as quickly as possible (ideally before departure) and maximising personal security within employers’ households.

*I advise them to try their best to learn Arabic before they migrate. In addition, I advise them to be very serious, and concentrate on their jobs. Arabs abuse you if you are panicking, but respect those who are very serious. ... If you fear them they want to abuse you in your every action. They don’t usually treat shy women in a good way. [Interview 1]*

*The maid shouldn’t sleep in the room which doesn’t have a lock. In my case I realized that my bedroom doesn’t have a lock the first day I arrived in the house. Then I told my employer that I can’t sleep there if she doesn’t fix the lock. … In my opinion it is too risky to sleep in a room which doesn’t have a lock. [FGD, 26]*

Despite difficulties accessing mobile phones, respondents nonetheless suggested that women should try to bring or obtain a phone to use in secret.

*If they can take the mobile from Ethiopia and take 50 Saudi birr to buy the SIM card from the airport. It is good to hide the phone. [Interview 5]*

Some women talked about good money management, recommending greater financial literacy, particularly planning how to save remittances. For instance, one woman explained:

*They [future migrants] will be successful if they get someone who saves their money properly. If they save the money, they can buy a car or build a house when they return home. For instance, I built this house with the money that my mother saved for me in a bank. [Interview 10]*

This woman also indicated the value of having a trusted relative who could ensure funds are saved or invested wisely, including advice to set up a formal bank account. There were warnings about migrants’ whose relatives spent all the money they had sent home, leaving them with nothing or even in debt on their return.

Returnees also stated that they believed in basic rights for domestic workers, which should be insisted on regardless of the legal framework in the destination country. Having gone through challenging or even traumatic experiences themselves, returnees conveyed the importance of resilience and self-worth for the next wave of migrants.

*They [new migrants] have to be aware of good and bad experiences that they will face when they arrive in the destination country. They should know their rights as well as their responsibilities before they migrate. [Interview 5]*

*When I was in Dubai my employer ordered me not to wear one specific skirt, she didn’t like it when I wore that, but I told her I liked the dress and there is no way that I won’t wear it. From that moment onwards she stopped complaining. I continued wearing it until I returned home. The migrant woman should be confident. [FGD, 25]*

## Discussion

Migration for domestic work is not uncommon in many resource-poor countries with high youth unemployment rates [22]. Thus, the fact that the Ethiopian women in our study consider migration for domestic work to be a good way to earn and secure a promising future is in keeping with the literature. Our study further confirms what other research has shown about the types of poor work conditions, health and safety hazards, and exploitation commonly encountered by migrants in domestic labour [8].

The main purpose of this study, however, was to look beyond risks faced during the migration process to identify practical and contextually appropriate messages and activities to strengthen the Freedom Fund’s Hotspot programme, which aims to increase individuals’ capacity to undertake safe migration. Our findings have been used to tailor the Freedom Fund’s interventions in the study area, and may also offer insights for safer migration interventions elsewhere.

We found that women and community members relied on family, neighbours, or local community contacts to determine labour migration options but did not always seek out or receive warnings about possible risks. Other studies in Ethiopia also found local people, particularly other returnees, are a key source of information for prospective migrants as they prepare for departure [23]. However, while close contacts were often trusted for migration-related planning information, women are not easily deterred by warnings about migration-related abuses and human trafficking. As also found elsewhere, stories of success and high incomes outweigh accounts of abuse by returning migrants or public information campaigns [12, 24].

On the other hand, migrants appreciated skills-based training such as how to conform to cultural expectations, operate domestic machinery, or communicate in rudimentary Arabic. For the most part, however, they relied on their own first-hand experiences in destination countries to develop strategies for overcoming adverse situations. Thus while returnees articulated recommendations for future migrants, they themselves did not appear to have had access to such specific advice.

This suggests that messages for prospective migrants must recognise that individuals are likely to have favourable and/or fatalistic attitudes towards their migration prior to departure. Awareness raising campaigns aiming to deter migration by instilling fears about human trafficking may not be successful. Rather, our study provides information on how returnees’ experiences can be constructively used to formulate a “harm reduction” approach. Using the data from migrants’ own narratives, we identify measures that may be protective for migrant domestic workers, and could be incorporated into skills-based trainings or more nuanced community education efforts. We characterised these “lessons learned” into four domains, depicted in Table 2: knowledge; skills; interpersonal characteristics; and access to resources.

**Table 2:** Risk avoidance factors

KNOWLEDGE	SKILLS
• Basic Arabic • Cultural expectations (clothing, gender relations) • Personal hygiene (using and disposing sanitary pads)	• Use of modern appliances and domestic products • Negotiating safety at work (e.g. bedroom locks)
INTERPERSONAL ATTRIBUTES	RESOURCES
• Confidence • Assertiveness • Obedience	• Phones and local sim cards • Contact details of agency, local Ethiopians, family • Leaving copy of contract with family

In terms of *knowledge*, returnees highlighted how familiarity with the destination country’s culture, language and behavioural expectations (local dress, use of menstrual products) could facilitate the transition to living in a foreign household. Worthwhile *skills* included use of modern cleaning and cooking appliances, and negotiating personal security. Adopting a confident and assertive demeanour, while remaining obedient, were *personal attributes* described by returnees as likely to earn employers’ respect and potentially diffuse conflict or deflect maltreatment. Finally, access to a mobile phone and sim card, and possession of contact details for employment agencies, local police, and/or other Ethiopians working nearby, were listed as important *resources* for domestic workers.

These four domains have implications for programming for safer migration. Skills-based training is already part of Ethiopian pre-departure programmes, and thus could be strengthened and expanded. Knowledge deemed useful by returnees could be integrated within skills-based training, with returnees more proactively involved in providing concrete examples of how information and resources proved strategic in times of crisis. Interestingly, our finding that an upfront, confident attitude can help protect migrants supports inclusion of pre-departure assertiveness. Assertiveness training has been used to reduce vulnerability to trafficking [25] and the International Organization for Migration recommends teaching self-sufficiency during pre-departure training [26]. However, this requires care, as it is also possible that in some situations assertiveness could incite abuse or mistreatment by employers.

Growing attention to human trafficking has led to a proliferation of pre-migration information and training programmes. As these interventions often involve substantial investments of both time and resources, it is important that activities draw on good, local evidence about people’s attitudes to migration and the factors that might protect people from exploitation and abuse. Incorporating robust findings about people’s knowledge, important skill sets and how they behaved with employers into safe migration interventions is likely to foster better pre-departure decision-making and preparation.

### Limitations

This study was conducted among a relatively small group of self-selected respondents who were willing to discuss their experiences as migrant domestic workers in the Gulf States. Respondents were referred from local authorities and then through personal contacts, e.g. “snowball sampling”. They are thus unlikely to be representative of the vast number of women who have undergone similar journeys. Although some respondents reported abuse and exploitation, our study may have under-recruited women with particularly negative experiences, including those traumatized by experiences of trafficking or severe abuse [16, 24]. Our focus on one part of Amhara region, known to be a significant “source” location for out-migration, means we cannot generalise our findings to other parts of the country with different ethnic, religious, and socio-economic profiles. However, our aim was to develop practical harm reduction messages in order to provide realistic advice to prospective migrants in the same geographical location, and for this purpose any positive bias or locally-specific insights among our respondents may have proved useful.

## Conclusion

There is little evidence on practices that foster safer, more successful labour migration, despite its acknowledged importance as a growing global issue. The numbers of young people seeking opportunities abroad continue to grow, as do concerns about associated exploitation, poor working conditions and concomitant psychological and physical morbidity. Ethiopia has a long history of out-migration and evidence shows that women seeking domestic work abroad comprise a large portion of current migrants.

With the spotlight now on decent work due to the 2030 Sustainable Development Goal 8.7 and the newly launched Alliance 8.7, which aims to “end child labour, forced labour, modern slavery and human trafficking” [27], evidence to inform anti-trafficking community-based programming has become even more in demand to protect those who are seeking to improve their lives by migrating for work. Globally, migrant workers are learning lessons about the opportunities and hazards of labour migration. We need to take the time and make the investments to benefit from their sometimes very hard-earned knowledge.
